# Screening tools to assess cancer-related financial toxicity: a scoping systematic review and thematic analysis protocol

**DOI:** 10.3389/fmed.2026.1723392

**Published:** 2026-03-11

**Authors:** Huan Li, Jing Luo, Shunlong Ou, Dongni Zheng, Qian Jiang

**Affiliations:** 1 Department of Pharmacy, Sichuan Clinical Research Center for Cancer, Sichuan Cancer Hospital and Institute, Sichuan Cancer Center, School of Medicine, University of Electronic Science and Technology of China, Chengdu, Sichuan, China; 2 Department of Pharmacy, The Second People’s Hospital of Yibin, Yibin, Sichuan, China; 3 Department of Pharmacy, Sichuan Clinical Research Center for Cancer, Sichuan Cancer Hospital and Institute, Sichuan Cancer Center, University of Electronic Science and Technology of China, Chengdu, Sichuan, China; 4 Department of Pharmacy, The Fifth People’s Hospital of Chengdu, Chengdu, Sichuan, China

**Keywords:** cancer, financial toxicity, protocol, systematic review, thematic analysis

## Abstract

**Introduction:**

Financial toxicity, stemming from high cancer treatment costs, compromises adherence, escalates caregiver burden, and induces psychological distress, ultimately hindering patient outcomes and exacerbating health inequities. Despite the development of various assessment tools, a consensus on how to uniformly measure financial toxicity remains elusive. This study aims to systematically analyze the similarities and differences among existing global financial toxicity assessment tools, identify key themes, and grade the evidence, thereby providing a theoretical foundation for developing a financial toxicity assessment tool tailored to the Chinese context.

**Methods:**

This review will conduct a comprehensive search of databases, including The Cochrane Library, PubMed, Embase, Web of Science, CNKI, Wanfang, VIP, CBM, grey literature, and health websites from various countries, to identify all tools assessing financial toxicity in cancer patients and their families. The search will cover the period from January 1, 2012, to January 1, 2026. Two reviewers will independently assess the included literature. In cases of disagreement, a third reviewer will adjudicate. Basic characteristics to be described will include the publishing institution, year of publication, country of origin, and applicable drug categories of the assessment tools. Key elements to be extracted and described will focus on dimensions of financial toxicity assessment, sources/evidence grading, and methodological processes, including methods and techniques. Subsequently, the thematic analysis method was adopted to integrate the evidence, extract key themes, and focus on the applicability of existing tools in the Chinese context. The integrated evidence will be evaluated using the GRADE-CERQual evidence quality assessment system to determine the credibility of the evidence for the applicability of existing tools.

**Ethics and dissemination:**

This study does not involve human or animal subjects, and ethical approval is not required. The results will be disseminated at various presentations and feedback sessions, in conference abstracts and manuscripts that will be submitted to peer-reviewed journals.

**Trial registration:**

PROSPERO registration number CRD42024546186.

## Highlights

- Financial toxicity (FT) is a deleterious consequence of cancer treatment characterized by financial hardship, impaired treatment adherence, and psychological distress. While numerous studies employ various FT assessment tools, a critical gap exists in the systematic evaluation of these tools’ validity, reliability, and applicability. This study provides the first comprehensive systematic review of tools used to assess financial toxicity.
- This research has two advantages: First, to critically evaluate the current landscape of global FT assessment tools through a comprehensive analysis of existing tools. This evaluation will focus on identifying the strengths, limitations, and methodological variations across different tools. Second, the study aims to synthesize current evidence regarding FT assessment into a systematic review that examines the existing assessment methodologies from the perspectives of tool development, methodology formation, risk stratification, and result presentation. This synthesis will provide a critical appraisal of the current state of FT measurement and inform the development of more effective tools.
- This research moves beyond the limitations of previous studies by (1) systematically comparing existing FT assessment tools within a standardized framework; (2) providing guidance and recommendations for the development of locally adapted financial toxicity assessment tools suitable for the Chinese setting.
- The study population was only for cancer patients, and financial toxicity assessment tools developed for other diseases could not be included in the study.
- The search strategy included Chinese and English databases, introducing a potential risk of language bias.

## Introduction

According to the latest global cancer data, new cancer cases are projected to reach 28 million, with 16.2 million deaths anticipated in 2024, highlighting cancer as a significant public health issue extending beyond its medical implications (1). As a cornerstone of oncologic treatment, pharmacotherapies have significantly enhanced patient quality of life and survival rates. However, the substantial financial investment required for research, development, and clinical trials, combined with the lengthy timelines inherent in bringing new oncology drugs to market, significantly contributes to their high cost (2, 3) Because of the high cost of cancer therapy, many cancer survivors are more likely to face substantial out-of-pocket healthcare expenditures and financial hardship, compared with persons without a history of cancer (4). Several studies have demonstrated that cancer survivors, irrespective of age, encounter treatment costs substantially exceeding 60,000 RMB, considerably surpassing the average annual patient income of 54,525 RMB (5, 6). Consequently, cancer patients experiencing considerable economic burden face an increased risk of treatment refusal, medication nonadherence, increased caregiver burden, and the development of mental health issues (7–9).

In response to the escalating costs of drug treatments for cancer patients, Bullock et al. (10). pioneered the conceptualization of cancer treatment costs as “financial toxicity” in 2012. In 2013, Zafar further refined this concept, noting that advances in cancer therapies have led to rising out-of-pocket costs for patients, potentially causing multifaceted and profound negative impacts on them (11).

To abrogate the impact of financial distress and minimize its potential to augment disparities, there is an urgent need for policymakers, researchers, and clinicians to accurately measure financial toxicity (12).

Currently, subjective scales constitute the predominant methodology for assessing financial toxicity. International research in this area has yielded notable results, leading to the development of several representative instruments, including the Socioeconomic Well-being Scale (SWBS) (13), Patient-Reported Outcome for fighting financial toxicity (PROFFIT) (14), the Subjective Financial Distress Questionnaire (SFDQ) (15), and the Comprehensive Score for financial toxicity based on the patient-reported outcome measure (COST) (16).

However, the utility of many of these scales, with the exception of COST, remains limited. Instruments such as economic strain and resilience in cancer (ENRICH) (17) and financial index of toxicity (FIT) (18) often suffer from a lack of large-sample validation studies to confirm their scientific rigor and generalizability. Furthermore, a major limitation is the prevailing focus on objective financial burden, resulting in insufficient attention paid to crucial subjective dimensions such as coping behavior modifications, levels of social support, and influence of family members (13, 16, 18).

In contrast, domestic research in China is nascent, characterized by a scarcity of indigenously developed tools. The majority of instruments are adaptations of established foreign scales, exemplified by the Chinese version of COST (translated by Luo Jing et al.) (19) and the head and neck cancer economic toxicity tool (translated by Jiang Hua et al.) (20). A significant hurdle for these adapted versions is the limited representativeness of their study samples and the lack of rigorous cultural adaptation and reliability testing within the unique context of the Chinese healthcare system.

Collectively, global financial toxicity assessment tools exhibit significant inconsistencies. These issues manifest as uneven quality, limited evaluative scope, unclear reliability and validity evidence, and substantial variance in measurement items and dimensional focus. Therefore, a systematic analysis and evidence synthesis are urgently needed to clarify the current landscape, identify core themes, and establish a robust foundation for future tool development.

This study aims to systematically review global financial toxicity assessment tools. The review will focus on the tools’ assessment properties, methodological frameworks, risk stratification approaches, and outcome presentation formats, analyzing the similarities and differences among various instruments. Building upon this analysis, a thematic analysis approach will subsequently be employed to explore the key influencing factors and thematic structures related to economic toxicity. Evidence levels associated with each identified theme will be rigorously graded. This comprehensive synthesis, when integrated with the specific socio-medical context of China, will subsequently provide the theoretical underpinning necessary for the future development of a localized, culturally appropriate economic toxicity assessment tool for the Chinese population.

## Objectives

This systematic review aims to critically evaluate financial toxicity assessment tools and provide evidence-based recommendations for developing economically applicable and locally adapted tools, particularly within the Chinese context.

## Methods and analysis

### Guideline

The review will be conducted according to Preferred Reporting Items for Systematic Reviews and Meta-Analyses (PRISMA) Protocols guidelines. The review protocol has been registered with the International Prospective Register of Systematic Reviews (PROSPERO 2021).

### Search strategy

The search strategy will combine key search terms and index terms relevant to qualitative studies using Boolean operators. The search will cover the period from January 1, 2012, to January 1, 2026. The database search was limited to articles published from 2012 onward, the year the concept of ‘financial toxicity’ was first introduced. A preliminary limited search will be conducted on PubMed, analyzing MeSH terms found in titles and abstracts, as well as indexing terms used to describe articles. This will inform the development of search strategies tailored to each database source. A second comprehensive search will utilize all identified keywords and index entries across relevant databases. Reference lists of all identified primary studies will be examined to identify additional studies relevant to this review. Previously published English and Chinese studies will be considered for inclusion in the database. A complete list of databases to be searched is detailed in Table 1.

**Table 1:** Search strategy.

Database	Retrieval strategy
CNKI	(Title: Cancer OR Neoplasm OR Cancer Patients OR Neoplasm Patients OR Cancer Family Members OR Neoplasm Family Members OR Cancer Caregivers OR Neoplasm Caregivers (fuzzy)) AND (Title: Tool OR Measurement OR Assessment OR Scale (fuzzy)) AND (Abstract: Economic OR Financial Toxicity OR Burden OR Stress (fuzzy))
Wan Fang	(Title: (Cancer OR Neoplasm OR Cancer Patients OR Neoplasm Patients OR Cancer Family Members OR Neoplasm Family Members OR Cancer Caregivers OR Neoplasm Caregivers) AND (Tool OR Measurement OR Assessment OR Scale) AND (Title OR Keywords: Economic OR Financial Toxicity OR Burden OR Stress))
VIP	((((((((((((Title or Keywords = Tumor OR Title or Keywords = Tumor Spread) OR Title or Keywords = Tumore) OR Title or Keywords = Tumors) OR Title or Keywords = Tumour) OR Title or Keywords = Cancer) OR Title or Keywords = Tumor Patient Family) OR Title or Keywords = Cancer Patient Family) OR Title or Keywords = Tumor Patient Caregiver) OR Title or Keywords = Cancer Patient Caregiver) AND (((Title or Keywords = Measurement OR Title or Keywords = Tool) OR Title or Keywords = Scale) OR Title or Keywords = Assessment)) AND (((Title or Keywords = Financial Toxicity OR Title or Keywords = Economic) OR Title or Keywords = Burden) OR Title or Keywords = Stress))
PubMed	((“scale”[Title/Abstract] OR “items”[Title/Abstract] OR “questionnaire”[Title/Abstract] OR “Instrument”[Title/Abstract] OR “evaluation”[Title/Abstract] OR “tool”[Title/Abstract]) AND (“financial stress”[MeSH Terms] OR “financial stresses”[Title/Abstract] OR “economic burden”[Title/Abstract] OR “financial burden”[Title/Abstract] OR “financial toxicity”[Title/Abstract] OR “financial challenge”[Title/Abstract] OR “financial pressure”[Title/Abstract] OR “financial strain”[Title/Abstract] OR “financial hardship”[Title/Abstract] OR “socioeconomic adversity”[Title/Abstract] OR “economic hardship”[Title/Abstract] OR “social difficulties”[Title/Abstract]) AND (“Tumors”[Title/Abstract] OR “Neoplasia”[Title/Abstract] OR “Cancer”[Title/Abstract] OR “malignant neoplasm”[Title/Abstract] OR “Malignancy”[Title/Abstract] OR “neoplasms”[MeSH Terms] OR “Oncology”[Title/Abstract]))
Embase	Refer to the PubMed search strategy
Web of Science	Refer to the PubMed search strategy
CBM	(“scale “[title: intelligent] OR” questionnaire “[title: intelligent] OR” evaluate “[title: intelligent] OR” measure “[title: intelligent] OR” framework “[title: intelligent] OR” tool “[title: intelligent]) AND (“economic toxicity” [title: intelligent] the OR “Economic pressure” [title: intelligent] OR “financial stress” [title: intelligent] OR “financial burden [title: intelligent]” OR “economic burden” [title: intelligent] OR “burden” [title: intelligent] OR [title: intelligent]) “pressure” (“tumor “OR [title: intelligent] “Cancer “[Title: Intelligent] OR” Cancer Patients “[Title: Intelligent] OR “Family Members of Cancer Patients “[Title: Intelligent] OR” Family Members of Cancer Patients “[Title: Intelligent] OR “Caregivers of Cancer Patients “[Title: Intelligent] OR “Cancer Patient Caregiver “[Title: Intelligence])
Cochrane	Refer to the PubMed search strategy

The search information database primarily includes: The Cochrane Library, PubMed, Embase, Web of Science, CNKI, Wan Fang Database, VIP Database, CBM databases, grey literature, and health websites from various countries. In addition, references from the relevant studies are manually filtered to identify additional studies.

### Inclusion and exclusion criteria

The inclusion criteria are based on compliance with the SPIDER framework. We will specifically apply the SPIDER framework to identify: Sample (S): cancer patients or family members of cancer patients; Phenomenon of interest (PI): Financial toxicity assessment tools; Design (D): Qualitative studies, mixed-methods studies, quantitative validation studies, and cross-sectional studies. Evaluation (E): Dimensions of financial toxicity assessment, sources/evidence grading, and methodological processes; Research type (R): Qualitative research.

The exclusion criteria include the following: (1) Secondary research, such as reviews, systematic reviews, and meta-analyses; (2) Articles focusing on non-cancer patients; (3) Articles not related to financial toxicity assessment tools; (4) Unavailable articles; (5) Duplicate articles; (6) Translated versions of financial toxicity assessment tools after localization.

### Data items

We will create a standardized data extraction table to collect relevant qualitative data from the included studies. We will extract: (1) Characteristics of the included tools methods: publisher, year, country, magazine name, website of relevant institutions, disease type, drug type, etc. (2) Key indicators: areas and indicators, sources and grading of evidence, types of included diseases, methods and tools of the production process, reliability, validity, responsiveness, interpretability, and the populations in which they were validated, etc. Two reviewers (HL and DN) independently extracted data from the articles back-to-back.

### Study selections

**Figure 1:**
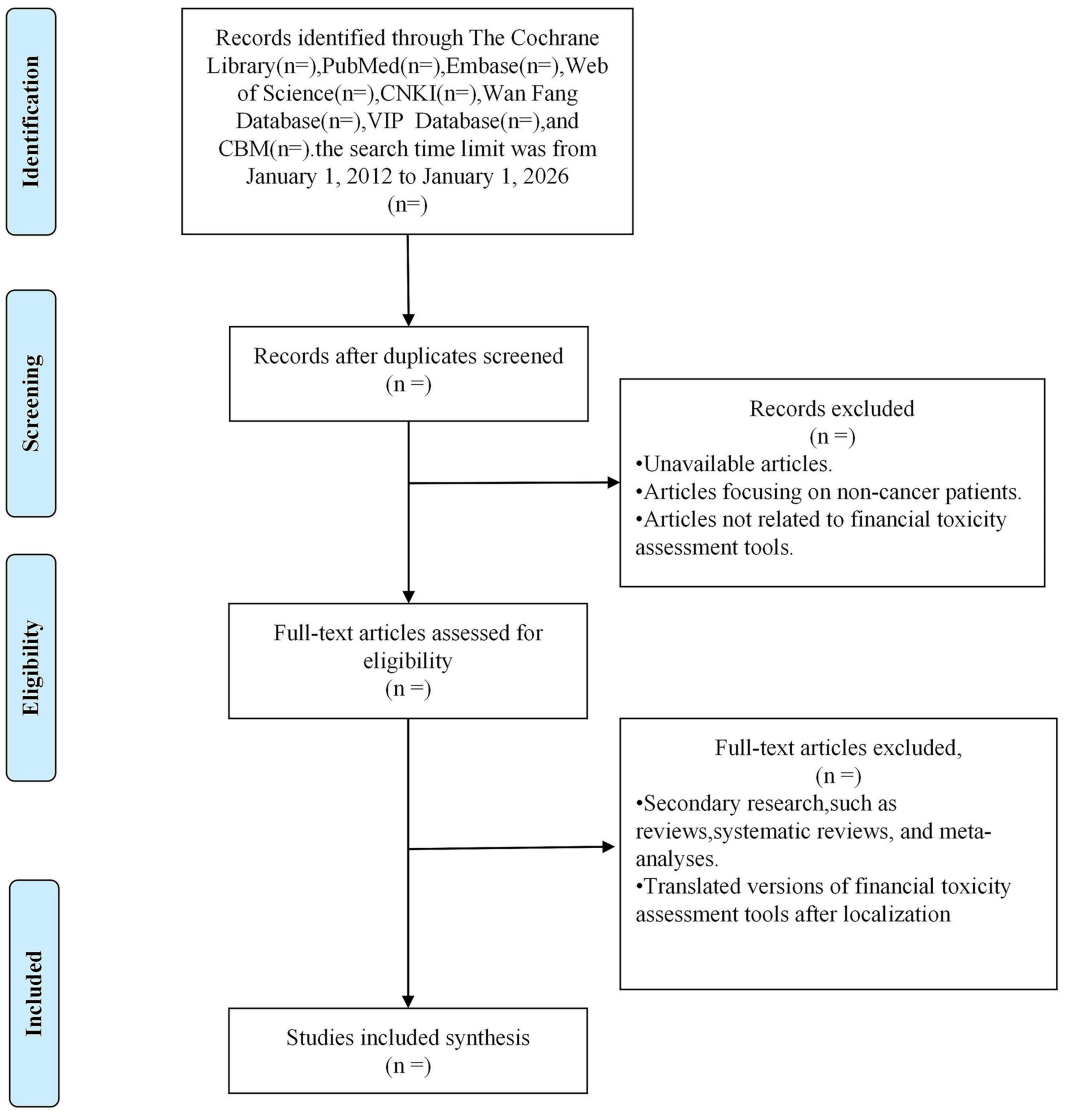
Planned flow diagram.

All retrieved studies will be curated and uploaded to Endnote 20. Duplicate studies will be excluded from the analysis. Two independent reviewers (HL and DN) will screen the titles and abstracts of the articles by the inclusion criteria. Because the search strategy includes both Chinese and English databases, we acknowledge the possibility of language bias. To minimize its impact, studies published in Chinese or English will be fully screened, and for potentially relevant studies published in other languages, professional translation support will be sought as needed to determine eligibility and extract data. Studies identified as potentially meeting the criteria or without abstracts will be retrieved for their full text. Two independent reviewers (HL and DN) will retrieve and evaluate the full text of the selected citations that meet the inclusion criteria. Full text that did not meet our inclusion criteria will be excluded, and the reasons for exclusion will be included in the final systematic evaluation report as an appendix. The included studies will undergo a rigorous screening process, and any differences between the two evaluators will be resolved by discussion. If no consensus is reached, a third reviewer (JL) will participate. The specific screening flow chart is shown in Figure 1.

### Risk of bias assessment

To ensure a rigorous evaluation of methodological bias in the included studies, we will conduct a formal Risk of Bias (RoB) assessment using the COSMIN Risk of Bias checklist (21). This tool is specifically designed for evaluating the methodological quality of studies developing or validating measurement instruments, which aligns with the aims of our review. The COSMIN checklist will allow us to systematically assess bias across key measurement properties, including content validity, structural validity, internal consistency, reliability, measurement error, hypothesis testing, and responsiveness. For each measurement property, COSMIN criteria will be applied to evaluate study design, sample adequacy, statistical methods, and reporting quality. Two reviewers will independently perform the RoB assessment following COSMIN scoring principles—rating each domain as “very good,” “adequate,” “doubtful,” or “inadequate.” Any discrepancies in ratings will be resolved through discussion or adjudication by a third reviewer to ensure consistency and methodological rigor. The COSMIN Risk of Bias tool is available in detail on the website: https://www.cosmin.nl/tools/checklists-assessing-methodological-study-qualities/.

### Methodological quality assessment

To assess the methodological rigor of included qualitative studies, two independent researchers (LH and DN) will employ the Critical Appraisal Skills Programme (CASP) tool (22). The CASP, designed for qualitative research appraisal, utilizes ten criteria to evaluate various aspects of study quality: research objectives, methodological appropriateness, research design, participant selection, data collection methods, data analysis techniques, interpretation of findings, research value, relationship between researchers and participants, and ethical considerations. Each criterion will be assessed as “yes,” “no,” or “unclear,” and an overall methodological quality rating of “high,” “medium,” or “low” will be assigned to each study. As the CASP tool does not recommend a scoring system, the overall ratings will be determined through consensus between the two researchers, based on a holistic evaluation of each criterion. Justifications for each rating will also be documented. In the case of mixed-methods studies, only the qualitative component will be subject to appraisal. The primary purpose of this quality assessment is to enhance the researchers’ comprehensive understanding of the included studies’ rigor and, consequently, to inform the classification of evidence. Therefore, the CASP appraisal results will not be used to weight the evidence synthesis. The CASP assessment tool is described in detail in Table 2.

**Table 2:** The CASP assessment tool.

CASP checklist: for qualitative research	
Section A: Are the results valid?	
Was there a clear statement of the aims of the research?	Yes No Cannot Tell
*CONSIDER:* *What was the goal of the research?* *Why was it thought important?* *Its relevance*	
Is a qualitative methodology appropriate?	Yes No Cannot Tell
*CONSIDER:* *If the research seeks to interpret or illuminate the actions and/or subjective experiences of research participants* *Is qualitative research the right methodology for addressing the research goal?*	
Was the research design appropriate to address the aims of the research?	Yes No Cannot Tell
*CONSIDER:* *if the researcher has justified the research design (e.g., have they discussed how they decided which method to use)*	
Was the recruitment strategy appropriate to the aims of the research?	Yes No Cannot Tell
*CONSIDER:* *If the researcher has explained how the participants were selected* *If they explained why the participants they selected were the most appropriate to provide access to the type of knowledge sought by the study* *If there are any discussions around recruitment (e.g., why some people chose not to take part)*	
Was the data collected in a way that addressed the research issue?	Yes No Cannot Tell
*CONSIDER:* *If the setting for the data collection was justified* *If it is clear how data were collected (e.g., focus group, semi-structured interview etc.)* *If the researcher has justified the methods chosen* *If the researcher has made the methods explicit (e.g., for interview method, is there an indication of how interviews are conducted, or did they use a topic guide)* *If methods were modified during the study. If so, has the researcher explained how and why* *If the form of data is clear (e.g., tape recordings, video material, notes etc.)* *If the researcher has discussed saturation of data*	
Has the relationship between researcher and participants been adequately considered?	Yes No Cannot Tell
*CONSIDER:* *If the researcher critically examined their own role, potential bias and influence during (a) formulation of the research questions (b) data collection, including sample recruitment and choice of location* *How the researcher responded to events during the study and whether they considered the implications of any changes in the research design*	
Section B: What are the results?	
Have ethical issues been taken into consideration?	Yes No Cannot Tell
*CONSIDER:* *If there are sufficient details of how the research was explained to participants for the reader to assess whether ethical standards were maintained* *If the researcher has discussed issues raised by the study (e.g., issues around informed consent or confidentiality or how they have handled the effects of the study on the participants during and after the study)* *If approval has been sought from the ethics committee*	
Was the data analysis sufficiently rigorous?	Yes No Cannot Tell
*CONSIDER:* *If there is an in-depth description of the analysis process* *If thematic analysis is used. If so, is it clear how the categories/themes were derived from the data* *Whether the researcher explains how the data presented were selected from the original sample to demonstrate the analysis process* *If sufficient data are presented to support the findings* *To what extent contradictory data are taken into account* *Whether the researcher critically examined their own role, potential bias and influence during analysis and selection of data for presentation*	
Is there a clear statement of findings?	Yes No Cannot Tell
*CONSIDER:* *If the findings are explicit* *If there is adequate discussion of the evidence both for and against the researcher’s arguments* *If the researcher has discussed the credibility of their findings (e.g., triangulation, respondent validation, more than one analyst)* *If the findings are discussed in relation to the original research question*	
Section C: Will the results help locally?	
How valuable is the research?	Yes No Cannot Tell
*CONSIDER:* *If the researcher discusses the contribution the study makes to existing knowledge or understanding (e.g., do they consider the findings in relation to current practice or policy, or relevant research-based literature)* *If they identify new areas where research is necessary* *If the researchers have discussed whether or how the findings can be transferred to other populations or considered other ways the research may be used*	

### Qualitative analysis

A qualitative analysis will be utilized to describe the basic characteristics of the included tools. These characteristics will include the country of publication, institution, year, applicable disease types, target populations, and outcome measures. The focused analysis will compare key elements, specifically the tools’ assessment domains, development methodology, and psychometric properties.

### Evidence synthesis and classification

For the evidence synthesis of this study, we will employ (23, 25) six-phase thematic analysis, explicitly applying it to compare and contrast included tools in terms of characteristics, assessment dimensions, psychometric properties, and contextual applicability. We will first extract data from eligible studies to generate descriptive and analytical insights, with emphasis on identifying elements misaligned with FT measurement in the Chinese context.

To develop tool adaptation-related themes, we will integrate inductive and deductive reasoning: deductive reasoning will be guided by a pre-established framework (rooted in financial toxicity theories and Chinese contextual adaptation priorities), while inductive reasoning will involve open coding of unanticipated findings and cross-study comparison to capture emerging sub-themes. This integration aims to produce contextually relevant interpretations for our research questions.

The synthesis will be facilitated by NVivo 15 software. To ensure the reliability and comprehensiveness of our findings, two researchers (LH and DN) will independently read the included literature multiple times. They will then systematically code the results and discussion sections of the papers. This systematic coding process will involve comparing concepts across different literature sources to ensure consistency and thoroughness. To minimize inter-researcher variability and address potential discrepancies, a process of recoding and categorizing content requiring further analysis will be undertaken. Through the naming, defining, and analysis of themes, new themes will be identified, and sub-themes will be consolidated to refine the coding and classification process.

Two researchers (LH and DN) will assess the confidence of the synthesized qualitative evidence using the GRADE-CERQual approach (24). CERQual evaluates each review finding across four domains: methodological limitations (informed by the CASP assessment), coherence, relevance, and adequacy of the data. Based on these domains, the reviewers will assign an overall confidence rating (high, moderate, low, or very low), with any disagreements resolved through discussion or consultation with a third reviewer (JL).

## Dissemination and implications

To strengthen the dissemination component of this study, the findings from the systematic review will be translated into actionable recommendations for researchers, clinicians, and policymakers, with specific consideration of the Chinese healthcare context. For researchers, the integrated evidence will identify methodological gaps in existing FT instruments and provide empirically grounded guidance for culturally appropriate tool development and psychometric validation. For clinicians, the synthesized results will support evidence-informed selection and implementation of assessment tools to improve early identification and management of FT in routine oncology practice. For policymakers, the qualitative insights and aggregated evidence will inform refinement of reimbursement policies, enhancement of financial protection mechanisms, and prioritization of support programs tailored to the economic burden faced by Chinese cancer patients. Study findings will be disseminated through peer-reviewed publications, academic conferences, and stakeholder-oriented briefs to facilitate their application in research, clinical decision-making, and policy development.
